# Supplementary material for: Content-rich biological network constructed by mining PubMed abstracts
Source: BMC Bioinformatics. 2004 Oct 8;5:147. doi: 10.1186/1471-2105-5-147 (PMC528731; doi:10.1186/1471-2105-5-147)
Supplement: Additional File 2 — The original results of the above study (non-essential files are deleted to keep the file size under the limit set by BMC bioinformatics). [file 1471-2105-5-147-S2.bz2 › chilibotAdditionalFile2/dip10/312ID11278571E1364/html/ZIPA_FTSZ.html]

 


 **ZIPA** and **FTSZ** 
  
Found 24 abstracts in PubMed, retrieved 10.  
 

 What does Google say? 
 PDF only 
| .edu only 

---

**Interactive relationship** (e.g. stimulation, inhibition, etc)

**Neutral relationship**- In vitro, wild type  **FtsZ**  interacted with  **ZipA**  with a high affinity in an enzyme linked immunosorbent assay.  Ref: 11278571 J Biol Chem, 2001
- The septal ring in Escherichia coli consists of at least nine essential gene products whose order of assembly resembles a mostly linear dependency pathway FtsA and  **ZipA**  directly bind  **FtsZ**  polymers at the prospective division site, followed by the sequential addition of FtsK, FtsQ, FtsL, FtsW, FtsI, and FtsN.  Ref: 11948172 J Bacteriol, 2002
- FtsA and  **ZipA**  localized into a division ring following the same pattern and time course as  **FtsZ** .  Ref: 12754232 J Bacteriol, 2003
- **ZipA** , a membrane protein involved in cell division that colocalizes with  **FtsZ** , was equally dynamic.  Ref: 11854462 Proc Natl Acad Sci U S A, 2002
- Characterization of  **FtsZ**  residues essential for the interactions with  **ZipA**  and with FtsA.  Ref: 11278571 J Biol Chem, 2001
- Genetic analysis of the Escherichia coli  **FtsZ** . **ZipA**  interaction in the yeast two hybrid system.  Ref: 11278571 J Biol Chem, 2001
- We have used polymerase chain reaction mediated random mutagenesis in the yeast two hybrid system to analyze this interaction and have identified residues within a highly conserved sequence at the C terminus of  **FtsZ**  as the  **ZipA**  binding site.  Ref: 11278571 J Biol Chem, 2001
- The cell division protein  **ZipA**   [ **ZIPA** ]  has an N terminal transmembrane domain and a C terminal globular domain that binds  **FtsZ** .  Ref: 12107152 J Bacteriol, 2002
- Structural evidence that the P Q domain of  **ZipA**  is an unstructured, flexible tether between the membrane and the C terminal  **FtsZ**  binding domain.  Ref: 12107152 J Bacteriol, 2002
- We have shown that a fusion, which encodes the last 70 residues of  **FtsZ**  in the two hybrid system, is sufficient for the interaction with FtsA and  **ZipA** .  Ref: 11278571 J Biol Chem, 2001
- **ZipA**  and FtsA are essential division proteins in Escherichia coli that are recruited to the division site by interaction with  **FtsZ** .  Ref: 11847116 EMBO J, 2002

**Non-interactive relationship** (e.g. studied together, co-existance, homology, etc.)

- MinCD prevented the localization of both  **FtsZ**  GFP and  **ZipA**  GFP, consistent with it preventing Z ring assembly.  Ref: 11673433 J Bacteriol, 2001
- Concentration and Assembly of the Division Ring Proteins  **FtsZ** , FtsA, and  **ZipA**  during the Escherichia coli Cell Cycle.  Ref: 12754232 J Bacteriol, 2003
- The recruitment of  **ZipA**  to the septum by  **FtsZ**  is an early, essential step in cell division in Escherichia coli.  Ref: 11278571 J Biol Chem, 2001
- These localization results support the model that E. coli cell division proteins assemble sequentially as a multimeric complex at the division site first  **FtsZ** , then FtsA and  **ZipA**  independently of each other, followed successively by FtsK, FtsQ, FtsL, FtsW, FtsI and FtsN.  Ref: 11703663 Mol Microbiol, 2001
- In Escherichia coli, at least nine essential gene products participate directly in septum formation FtsA, FtsI, FtsL, FtsK, FtsN, FtsQ, FtsW,  **FtsZ**  and  **ZipA** .  Ref: 11703663 Mol Microbiol, 2001
- Mutations surrounding Asp 373 differentially affected the interactions of  **FtsZ**  with  **ZipA**  and FtsA, indicating that these proteins bind the C terminus of  **FtsZ**  differently.  Ref: 11278571 J Biol Chem, 2001
- Consistent with this, we found that an  **FtsZ**  mutant unable to interact with both  **ZipA**  and FtsA was unable to assemble into Z rings.  Ref: 11847116 EMBO J, 2002
- **ZipA**  and FtsA are recruited independently to the  **FtsZ**  cytokinetic ring Z ring and are essential for cell division of Escherichia coli.  Ref: 12634424 Proc Natl Acad Sci U S A, 2003
- The concentration of the cell division proteins  **FtsZ** , FtsA, and  **ZipA**  and their assembly into a division ring during the Escherichia coli B r K cell cycle have been measured in synchronous cultures obtained by the membrane elution technique.  Ref: 12754232 J Bacteriol, 2003
- The results imply that association of both FtsA and  **ZipA**  with  **FtsZ**  polymers is needed for further maturation of the nascent organelle.  Ref: 11948172 J Bacteriol, 2002
